# Rural, urban and suburban differences in the prevalence of metabolic syndrome in individuals aged ≥50 years in Northwest China

**DOI:** 10.3389/fpubh.2025.1589196

**Published:** 2025-07-16

**Authors:** Yang Jiao, Chunhong Zhang, Jie Ming, Shaoyong Xu, Yangwei Wang, Xiaoli Yao, Aihua Jia, Hui Li, Jing Sui, Jianjun Qin, Wenjie Li, Haixiong Zhang, Xin Zhao, Xuan Xie, Qiuhe Ji

**Affiliations:** ^1^Department of Endocrinology, Second Affiliated Hospital of Xi’an Jiaotong University, Xi’an, China; ^2^Department of Endocrinology, Xijing Hospital, Xi’an, China; ^3^Department of Endocrinology, Shaanxi Provincial People’s Hospital, Xi’an, China; ^4^Department of Endocrinology, The First Affiliated Hospital of Xi’an Jiaotong University, Xi’an, China; ^5^Department of Medicine, Staff Hospital of Shaanxi Blower (Group) Co., Ltd., Xi’an, China; ^6^Department of Endocrinology, 521 Hospital of Norinco Group, Xi’an, China; ^7^Department of Endocrinology, Xi’an Aerospace General Hospital, Xi’an, China

**Keywords:** metabolic syndrome, type 2 diabetes, rural, suburban, urban

## Abstract

**Background:**

Metabolic syndrome (MetS) is a significant risk factor for type 2 diabetes and cardiovascular disease. The aim of this study was to identify the characteristics of MetS in northwest China.

**Methods:**

Three thousand and one adults were included (1,915 females, 1,086 males). The prevalence of MetS analysis was stratified according to gender, age and the region of residence.

**Results:**

MetS prevalence in females and males was 49.7 ± 9.8% and 32.0 ± 9.0%, respectively (*p* < 0.001). MetS prevalence in females increased with age and was greater in rural (*n* = 217) females (53.6%) compared to urban (*n* = 754) (45.5%) or suburban (*n* = 818) females (52.0%) (*p* = 0.003). Regression analysis revealed that rural region, age, half-meat and half-vegetable dietary style, never dieted, weight increase in the previous year and a family history of high blood pressure were independent risk factors for the development of MetS, particularly in women aged ≥50 years.

**Conclusion:**

MetS prevalence in women was greater than for men, increased with age and occurred more frequently in rural compared to urban and suburban females.

## 1 Introduction

Metabolic syndrome (MetS) is associated with an increased risk of type 2 diabetes, cardiovascular disease and overall mortality (1). In particular, older adult women are at heightened risk, with several studies showing a strong correlation between MetS and the subsequent development of type 2 diabetes and cardiovascular disease (2, 3). A national Chinese survey of 15,540 participants reported an age-standardized MetS prevalence of 17.8% in women and 9.8% in men (4). One significant factor contributing to MetS in women aged ≥50 years was the menopause. Hormonal changes, including decreased estrogen and increased testosterone have been identified as key contributors to MetS development during and after menopause (5–7).

While MetS has been widely studied in general populations, regional and residential distinctions – particularly among rural, suburban, and urban populations – remain underexplored. Emerging evidence suggests that these differences may significantly affect MetS prevalence and progression due to variations in socioeconomic status, lifestyle, health literacy and access to healthcare. In particular, older adult women in rural areas may face elevated risks, yet they have been underrepresented in existing studies. For example, compared to men, women show lower rates of MetS resolution when overweight, while factors such as low income and current employment influence MetS differently across genders (8). Historically, rural areas in China have exhibited lower MetS prevalence compared to urban regions. However, recent studies indicate a significant rise in MetS cases in rural communities. A study in rural northeast China found MetS prevalence rates comparable to, or even exceeding, those in some urban areas, with rates reaching 24.2% in 2014, up from 4.3% in 2002 (9). In rural Xinjiang, a multi-ethnic region, age-standardized MetS prevalence ranged from 14.43 to 26.50%, depending on the diagnostic criteria use (10). Suburban areas, often representing transitional zones between rural and urban settings, show intermediate MetS prevalence rates. A study that compared two types of urbanization in China – rural-to-urban migration and *in situ* urbanization – found varying impacts on metabolic health. In rapidly urbanizing areas, the prevalence of diabetes, obesity and non-alcoholic fatty liver disease increased significantly (11). Proposed contributing factors were that transition from agricultural to industrial jobs reduced physical activity, adoption of urban dietary patterns, sedentary behaviors and improved healthcare infrastructure compared to rural areas, but may still lag behind urban centers. Urban residents in China have traditionally exhibited higher MetS prevalence due to lifestyle factors such as higher consumption of high-calorie processed foods, sedentary occupations and reduced physical activity levels, but better access to healthcare services has facilitated the diagnosis and management of MetS components. Studies have reported the prevalence of MetS among urban residents ranging from 17.91 to 35.10% in men and 26.04 to 32.50% in women (12). Lifestyle factors such as diet, physical activity and substance use also play a critical role in the risk of developing MetS, with regional differences in lifestyle habits influencing its prevalence and characteristics, and also in other countries (13–16).

Despite this growing body of research, most studies have focused on urban populations, leaving rural women underrepresented. Recent evidence has challenged earlier assumptions, indicating a higher prevalence of MetS in rural areas – possibly driven by dietary transitions and socioeconomic changes. Rural women, in particular, often experience lower health literacy, poorer dietary habits and reduced participation in preventive health behaviors such as diet control. The data derived from the Community-Based Management of Diabetes in the Elderly (CBMDE) project, an epidemiological and prospective cohort study, which employed a community-based multi-stage, stratified, cluster sampling method whose first step, covered between June 2014 and September 2016, was analyzed in order to achieve the prevalence data of diabetes in the northwest, representing Shaanxi Province in China. To address an existing research gap, we analyzed data from the CBMDE survey, focusing on individuals aged ≥50 years in northwest China. The present study was centered on a high-risk yet under-studied group – rural women over 50 years old – highlighting unique risk factors and evolving epidemiological patterns. Our findings contribute not only to the academic literature on MetS distribution but also provide practical guidance for designing targeted public health interventions and informing regional and national health policy.

## 2 Methods

### 2.1 Study population

This was a cross-sectional epidemiology investigation based on community populations and is part of the CBMDE project in Xi’an. The CBMDE project is a prospective, community-based, multi-stage, stratified and cluster-sampled epidemiological and prospective cohort study that is focused on diabetes. Its aims were to select a representative sample of middle-aged and older adults (aged ≥50 years) with diabetes from both urban and rural communities in Xi’an and its surrounding areas for long-term follow-up and management. The goals were to investigate the risk factors and preventive strategies related to diabetes and other metabolic disorders in this population. The survey was conducted from June 2014 to September 2016, in order to determine the prevalence data of diabetes and MetS in northwest China, mainly the Shaanxi Province (17). This study was reviewed and approved by the Ethics Committee of Xijing Hospital (approval number 20130925-8). All participants signed informed consent before being included in the study.

### 2.2 Data collection

The definitions of urban, rural and suburban areas refers to the “Regulation on Classification of Urban and Rural Areas for Statistics” issued by the National Bureau of Statistics of China (18). The questionnaire was completed by study participants, assisted by an inquirer and included demographic data, current disease information, family history of disease and life-style information. The data collection was conducted as follows:

#### 2.2.1 Determination of the data acquisition areas

In the first stage, a selection of urban, suburban and rural areas was performed. Four urban districts, three suburban areas and two rural regions (one affluent and one economically disadvantaged) in Xi’an and its surrounding areas were selected. This stage was non-random. In the second stage specific communities and villages were selected. Within the selected urban, suburban and rural areas, seven communities and seven villages were chosen, including four urban communities, three suburban communities, one affluent large village and six smaller or less affluent villages. This stage was also non-random.

#### 2.2.2 Data collection from the participants living in the areas determined in stage 1 and 2

In the third stage a stratified cluster sampling within communities or villages was conducted. In each selected community or village, stratification was carried out based on the local population distribution. Within each stratum, clusters (residential buildings or natural villages) were randomly selected using a simple random sampling method. The study population included individuals aged ≥50 years old.

#### 2.2.3 Specific data collection regarding health status

Physical examinations included height (standing barefoot), weight, waist circumference (standing feet separated) and the circumference of the hip (at the level of the greatest protrusion of the buttocks, standing feet separated) were measured when the subjects wore underwear; the average blood pressure of 2 measurements taken from the right arm in a resting state was calculated. Venous blood was collected in 5 mL aliquots after the subjects had fasted overnight. Plasma concentrations of glucose and lipids were assessed on the same day at each sub-center using a standardized automated analyzer. The diagnostic criteria for MetS that originally followed the 2013 Chinese Diabetes Society criteria (19) were again analyzed in the present study according to the International Diabetes Federation (IDF) criteria (20): central obesity was defined as a circumference of the waist ≥80 cm for women and ≥90 cm for men. In addition, 2 of various criteria were taken into consideration including: (1) a triglyceride level ≥1.7 mmol/L or the participant was receiving specific treatment for this lipid abnormality; (2) a decrease in high-density lipoprotein cholesterol (HDL-C) concentrations: men a value <1.03 mmol/L, women a value <1.29 mmol/L, or given treatment; (3) raised systolic blood pressure ≥130 mmHg, diastolic blood pressure ≥85 mmHg or had received therapy for hypertension; (4) a fasting plasma glucose (FPG) concentration ≥5.6 mmol/L or the participant was diagnosed as having type 2 diabetes. Health status was not included as an inclusion criterion, as this was a cross-sectional study that aimed to estimate the prevalence of MetS in the general population. Applying health-related restrictions would have biased the results and affected the representativeness of the findings.

### 2.3 Statistical analyses

All data were independently entered by two researchers and verified for consistency. The final dataset was constructed using EpiData version 3.1 and statistical analyses were performed using SPSS version 18.0 (IBM Corp., Armonk, NY, USA). A two-sided *p*-value < 0.05 was considered to be statistically significant.

Descriptive statistics were used to summarize the characteristics of study participants. Categorical variables are expressed as frequencies and percentages, and continuous variables as the mean ± standard deviation (SD) or median with interquartile range (IQR), depending on the nature of the distribution. Group comparisons for categorical variables were conducted using the Chi-squared (*χ*^2^) test. For continuous variables, one-way analysis of variance (ANOVA) was applied when normality (Shapiro–Wilk test) and homogeneity of variance (Levene’s test) assumptions were met. Non-parametric comparisons were performed using the Kruskal–Wallis test when these assumptions were violated.

To assess factors independently associated with the prevalence of MetS, multivariable logistic regression analysis was conducted. The dependent variable was the presence or absence of MetS (binary outcome). Independent variables were selected based on prior literature and biological plausibility, and included: age group (50–59, 60–69, ≥70 years); the place of residence (rural, urban, suburban); educational attainment (primary school or below, junior high school, high school or above); household income (≤¥10,000 [$ ≈ 1,400], ¥10,000–30,000 [$ ≈ 1,400–4,200], >¥30,000 [$ ≈ 4,200]/year); delayed menarche (yes/no); dietary pattern (meat-based, mixed, vegetable-based), salt intake (high, normal, low); type of meat consumed (fatty, mixed, lean); dietary control (yes/no); sleep duration (≤6 h, 6–8 h, >8 h); sedentary time (≤7 h/day, 7–9 h/day, >9 h/day); body weight change over the past year (increased, decreased, unchanged); and family history of diabetes, hypertension and dyslipidemia (all yes/no). Categorical variables were coded using dummy variables. Continuous metabolic parameters such as waist circumference and blood pressure, used to define MetS, were not included as predictors in the regression model. Associations were reported as odds ratios (ORs) with 95% confidence intervals (CIs) and Wald tests were used to evaluate statistical significance between individual predictors.

Missing data were excluded from the analysis if determined to be non-ignorable (e.g., lifestyle variables prone to recall bias). No data imputation was performed.

Sample size estimation was based on an assumed diabetes prevalence of 25%, with a 2.5% margin of error and 95% confidence level. Adjusting for an expected response rate of 85% and applying a design effect of 2.0 due to multistage sampling, the target sample size was 2,800. A total of 3,119 participants were ultimately enrolled. Given the higher-than-expected observed MetS prevalence (49.7% in females, 32.0% in males), the study was deemed adequately powered to detect significant associations for both the estimation and regression analyses.

## 3 Results

**Figure 1:**
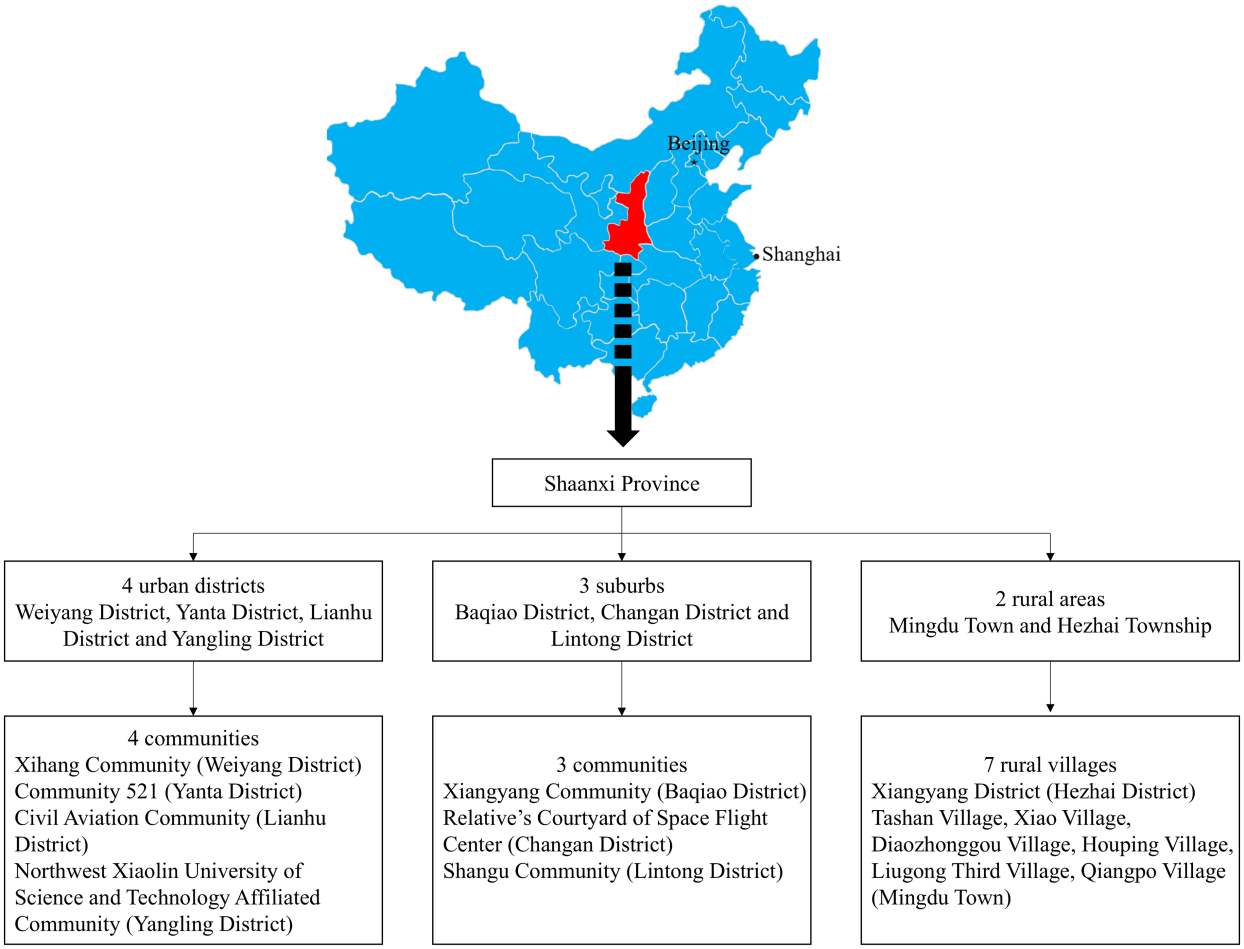
Study sampling procedure and location of Shaanxi province in mainland China. Stage 1: Selection of urban, suburban and rural areas. A total of 4 urban districts, 3 suburban areas and 2 rural areas (including one affluent rural area and 1 underdeveloped rural area) were selected. Stage 2: Within the selected urban, suburban and rural areas, specific communities and villages were further selected. A total of 7 communities and 7 villages were included, consisting of 4 urban communities, 3 suburban communities, 1 affluent village (large village) and 6 underdeveloped villages (small to medium-sized villages).

In the first stage of the study, 2 rural areas, 3 suburbs and 4 urban districts were selected. In the second stage, 7 representative rural villages and 7 communities were chosen from the areas mentioned above, including 1 large and 6 small-to-medium rural villages, and 3 suburban and 4 urban communities (Figure 1). In the third stage, the population was chosen using stratified random sampling by community, buildings and the living unit in each community or village. The response rate was 75.45% and 3,119 participants aged ≥50 years were selected from which 3,001 with complete data were finally included in the analyses. In total, 118 participants lacked key data on FPG, 2-h postprandial plasma glucose and glycated hemoglobin, and were excluded from further analyses.

### 3.1 General information about the study participants

The study involved a cohort of 3,001 participants, aged ≥50 years who had full physical examination and complete laboratory data, and was comprised of 1,915 females and 1,086 males (Table 1).

**Table 1:** General clinical characteristics of the sample population.

Index	Total *N* = 3,001	Women *N* = 1,915	Men *N* = 1,086	*p*-value
Age (years)	62.8 ± 7.9	62.4 ± 7.6	63.5 ± 8.1	<0.001
Body mass index (kg/m^2^)	25.0 ± 3.5	24.9 ± 3.7	25.1 ± 3.2	0.074
Waist circumference (cm)	87.3 ± 9.8	86.1 ± 9.7	89.5 ± 9.7	<0.001
Hip circumference (cm)	97.5 ± 10.4	97.3 ± 11.6	97.7 ± 7.8	0.376
Systolic blood pressure (mmHg)	128.7 ± 18.6	128.7 ± 19.1	128.7 ± 17.9	0.098
Diastolic blood pressure (mmHg)	79.3 ± 10.6	78.6 ± 10.3	80.5 ± 11.0	<0.001
FPG (mmol/L)	5.9 ± 1.9	5.8 ± 1.8	6.0 ± 2.0	0.029
Blood triglycerides (mmol/L)	1.8 ± 1.0	1.8 ± 1.0	1.7 ± 1.0	0.054
Blood HDL-C (mmol/L)	1.3 ± 0.4	1.4 ± 0.4	1.2 ± 0.3	<0.001

Data are presented as the mean ± SD.

FPG, fasting plasma glucose; HDL-C, high-density lipoprotein cholesterol.

### 3.2 The prevalence of MetS according to gender

**Figure 2:**
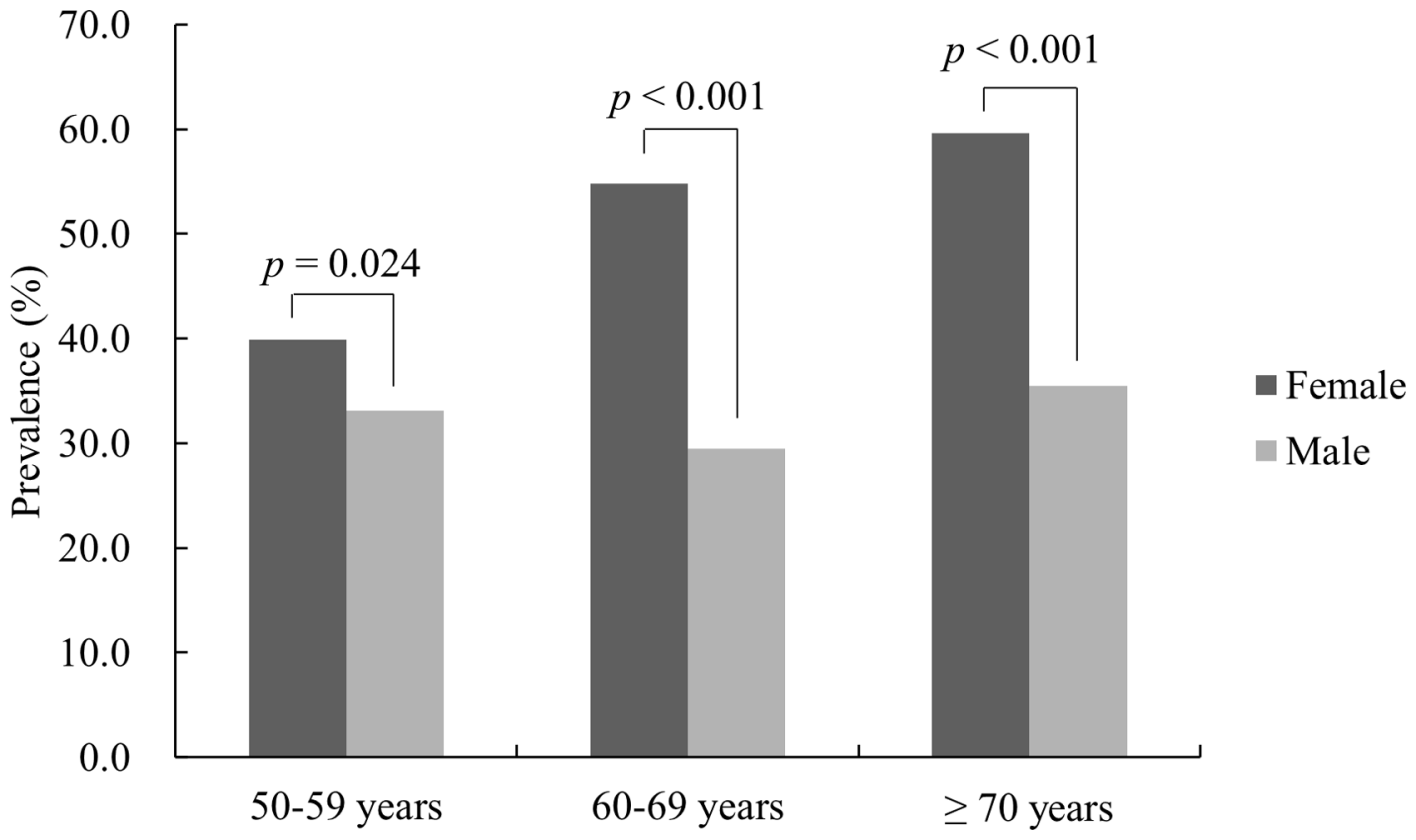
MetS prevalence in females and males aged ≥50 years stratified by age.

According to the diagnostic criteria of the IDF, the prevalence of MetS was greater in women aged ≥50 years than in men (49.7 ± 9.8% *vs.* 32.0 ± 9.0%, *p* < 0.001). Similarly, using IDF diagnostic criteria, the prevalence rate for MetS in females was greater than for men in the 50–59 years old group (39.9 ± 4.7% *vs.* 33.1 ± 10.3%, *p* = 0.024), the 60–69 years group (54.8 ± 5.2% *vs.* 29.5 ± 6.3%, *p* < 0.001) and the ≥70 years group (59.6 ± 7.0% *vs.* 35.5 ± 9.6%, *p* < 0.001). There was a difference in prevalence among female age groups (*p* < 0.001), which increased with age. There was also a difference for men aged 50–59 years, 60–69 years and ≥70 years (*p* = 0.035), but no significant increase in prevalence with age was found (Figure 2).

### 3.3 Regional differences in the prevalence of MetS in women aged ≥50 years

According to IDF criteria, MetS prevalence was greater in rural region females than in urban region and suburban region females 53.6% *vs.* 45.5 and 52.0%, (*p* = 0.003). The same trend was also found for the 50–59 and ≥70 year age groups. MetS prevalence increased with age in urban and suburban areas, but in rural areas this trend was only detected for ages up to 69 years, with, interestingly, less prevalence in over 70-year-old wives (Table 2).

**Table 2:** Prevalence of MetS in females over 50 stratified by age and the region of residence.

Variable	Rural		Urban		Suburban		*p-*value^†^
	*n*/*N*	%	*n*/*N*	%	*n*/*N*	%	
Total	184/217	53.6	343/754	45.5	425/818	52.0	0.003
50–59 years	65/139	46.8	127/337	37.7	113/288	39.2	0.045
60–69 years	84/140	60.0	150/294	51.0	209/375	55.7	0.096
≥70 years	35/64	54.7	66/123	53.7	103/155	66.5	0.005
*p*-value^‡^		<0.004		<0.001		<0.001	

^†^Represents the comparison of prevalence in the same age group in different areas; ^‡^Represents the comparison of prevalence in different age groups in the same area.

HDL-C, high-density lipoprotein cholesterol; MetS, metabolic syndrome.

Comparing the prevalence of single MetS components, central obesity and hypertension rates were highest in rural areas, but hyperglycemia and decreased HDL-C values were lowest. In urban females, hypertriglyceridemia occurred most frequently but the central obesity prevalence was lowest. Suburban females had the most decreased HDL-C values and highest prevalence of hyperglycemia, but the lowest hypertriglyceridemia and hypertension rates (Table 3).

**Table 3:** Frequency of MetS components in females aged ≥50 years stratified by their region of residence.

Component	Rural		Urban		Suburban		*p*-value			
	*N* = 343		*N* = 754		*N* = 818					
	*n*	%	*n*	%	*n*	%	Overall	Rural *vs.* urban	Rural *vs*. suburban	Urban *vs.* suburban
Central obesity	289	84.8	520	69.5	653	80.8	<0.001	<0.001	0.022	<0.001
Hypertriglyceridemia	166	48.4	372	49.3	343	41.9	<0.001	0.752	0.003	<0.001
Decreased HDL-C level	113	32.9	324	43.0	375	45.8	<0.001	<0.001	<0.001	0.168
Hypertension	234	68.4	441	58.5	448	54.8	<0.001	<0.001	<0.001	0.078
Hyperglycemia	91	26.5	275	36.5	343	41.9	<0.001	<0.001	<0.001	0.009

HDL-C, high-density lipoprotein cholesterol; MetS, metabolic syndrome.

### 3.4 Demographic and lifestyle characteristics of females in different regions

The ratio of a low level of education plus a low family income of women aged ≥50 years living in a rural region was greater than for urban and suburban women (50.7% *vs.* 19.0 and 25.3% for low education level, 65.4% *vs.* 21.9 and 13.4% for low family income, *p* < 0.001). The rural population experienced delayed menarche and earlier menopausal ages compared to urban and suburban females (16.5 ± 2.2 *vs.* 14.9 ± 2.1 and 15.2 ± 2.0 for menarche age, *p* < 0.001; 48.5 ± 4.5 *vs.* 49.6 ± 3.9 and 49.1 ± 4.2 for menopause age, *p* = 0.001). The proportion of menarches occurring later was higher in rural women aged ≥50 years than in urban and suburban women (12.1% *vs.* 4.0 and 4.8%, respectively, *p* < 0.001).

Compared to suburban and urban women, rural women were more likely to have a diet mainly based on vegetables (86.2% in rural areas *vs.* 56.1% in urban areas and 56.6% in suburban areas, *p* < 0.001), meals containing high salt (26.1% in rural areas *vs.* 16.7% in urban areas and 18.7% in suburban areas, *p* < 0.001) and moderately fatty meat (64.7% in rural areas *vs.* 48.2% in urban areas and 41.2% in suburban areas, *p* < 0.001). However, only approximately 19.2% of rural women tried diet control compared to a greater proportion of urban and suburban women (45.0 and 40.3%, *p* < 0.001).

Women who live in rural regions exhibited different work and rest schedules, since they had more sleep time (*p* < 0.001), while the sedentary time which rural women aged ≥50 years spent was equivalent to that of urban women. Regarding weight, about 25.2% of rural women had gained weight in the past year before the investigation, which was essentially higher than for the 17.9% of suburban and 17.6% of urban females (*p* = 0.009).

Interestingly, in rural females aged ≥50 years the family history of type 2 diabetes (8.2% *vs.* 21.1 and 21.0%, *p* < 0.001), hypertension (34.7% *vs.* 46.9 and 45.4%, *p* < 0.001) and dyslipidemia (5.0% *vs.* 21.5 and 20.7%, *p* < 0.001) were all lower compared to urban and suburban females. Compared to those living in urban and suburban regions, rural women had a greater waist circumference (87.7 ± 8.9 *vs.* 84.1 ± 9.7 and 87.2 ± 10.1 cm, *p* < 0.001), systolic blood pressure (133.3 ± 21.6 *vs.* 130.2 ± 18.8 and 125.7 ± 17.7 mmHg, *p* < 0.001), diastolic blood pressure (82.2 ± 10.0 *vs.* 78.8 ± 10.6 and 77.0 ± 9.7 mmHg, *p* < 0.001), triglyceride level (1.9 ± 1.3 *vs.* 1.8 ± 0.9 and 1.7 ± 1.0, *p* = 0.005) and the lowest plasma glucose concentration (5.5 ± 1.9 *vs.* 5.9 ± 2.0 and 5.8 ± 1.5 mmol/L, *p* = 0.001), findings which are consistent with the data reported for the highest abdominal obesity, hypertension and hypertriglyceridemia prevalence (Table 4).

**Table 4:** Characteristics of females aged ≥50 years from different regions of residence.

Variable	Rural	Urban	Suburban	*p*-value			
Number	343	754	818	Overall	Rural *vs.* urban	Rural *vs.* suburban	Urban *vs.* suburban
Age, years	62.4 ± 7.9	61.9 ± 7.5	63.1 ± 7.7	0.008	0.305	0.164	0.002
Education level, *n* (%)	*n* = 341	*n* = 748	*n* = 813				
Elementary school or below	173 (50.7)	142 (19.0)	206 (25.3)	<0.001	<0.001	<0.001	0.003
Secondary school	165 (48.4)	493 (65.9)	534 (65.7)	<0.001	<0.001	<0.001	0.957
College or above	3 (0.9)	113 (15.1)	73 (9.0)	<0.001	<0.001	<0.001	<0.001
Yearly family income, *n* (%)	*n* = 332	*n* = 684	*n* = 729				
≤¥10,000 (≤$1,400$)	217 (65.4)	150 (21.9)	98 (13.4)	<0.001	<0.001	<0.001	<0.001
¥10,000–30,000 ($1,400–4,200$)	98 (29.5)	192 (28.1)	297 (40.7)	<0.001	0.657	<0.001	<0.001
≥¥30,000 (≥$4,200$)	17 (5.1)	342 (50.0)	334 (45.8)	<0.001	<0.001	< 0.001	0.122
Age of menarche, years	16.5 ± 2.2	14.9 ± 2.1	15.2 ± 2.0	<0.001	<0.001	<0.001	0.006
Menstruation delay (>18 years old), *n* (%)	41/339 (12.1)	29/730 (4.0)	38/796 (4.8)	<0.001	<0.001	<0.001	0.456
Age of menopause, years	48.5 ± 4.5	49.6 ± 3.9	49.1 ± 4.2	0.001	<0.001	0.032	0.030
Menopause, *n* (%)	329/343 (95.9)	714/754 (94.7)	781/818 (95.5)	0.617			
Premature menopause (≤40 years old), *n* (%)	22/324 (6.8)	26/688 (3.8)	33/755 (4.4)	0.095			
Dietary style, *n* (%)	*n* = 333	*n* = 742	*n* = 738				
Meat-based	5 (1.5)	20 (2.7)	19 (2.6)	0.512			
Half and half	41 (12.3)	306 (41.2)	301 (40.8)	<0.001	<0.001	<0.001	0.874
Vegetable-based	287 (86.2)	416 (56.1)	418 (56.6)	<0.001	<0.001	<0.001	0.834
Dietary salt consumption, *n* (%)	*n* = 333	*n* = 742	*n* = 738				
High-salt	87 (26.1)	124 (16.7)	138 (18.7)	0.001	<0.001	0.006	0.340
Normal-salt	145 (43.5)	364 (49.1)	304 (41.2)	0.004	0.064	0.504	0.001
Low-salt	101 (30.3)	254 (34.2)	296 (40.1)	0.004	0.233	0.003	0.021
Dietary meat consumption, *n* (%)	*n* = 323	*n* = 734	*n* = 728				
Fatty meats	6 (1.9)	19 (2.6)	12 (1.6)	0.451			
Moderately fatty meat	209 (64.7)	354 (48.2)	300 (41.2)	<0.001	<0.001	<0.001	0.007
Lean meats	108 (33.4)	361 (49.2)	416 (57.1)	<0.001	<0.001	<0.001	0.002
Dietary control, *n* (%)	*n* = 333	*n* = 742	*n* = 734				
Ever	64 (19.2)	334 (45.0)	296 (40.3)	<0.001	<0.001	<0.001	0.074
Never	269 (80.8)	408 (55.0)	438 (59.7)				
Sleep duration, h	8.5 ± 1.2	8.2 ± 3.0	7.7 ± 1.7	<0.001	0.102	<0.001	<0.001
Sleep duration, *n* (%)	*n* = 343	*n* = 754	*n* = 818				
≤6 h	27 (7.9)	94 (12.5)	190 (23.2)	<0.001	0.029	<0.001	<0.001
6–8 h	135 (39.4)	399 (52.9)	394 (48.2)	<0.001	<0.001	0.007	0.062
>8 h	181 (52.8)	261 (34.6)	234 (28.6)	<0.001	<0.001	<0.001	0.011
Sedentary time, h	8.5 ± 3.9	8.7 ± 4.1	10.0 ± 4.8	<0.001	0.700	<0.001	<0.001
Sedentary time, *n* (%)	*n* = 332	*n* = 740	*n* = 757				
≤7 h/day	140 (42.2)	305 (41.2)	243 (32.1)	<0.001	0.789	0.002	<0.001
7–9 h/day	45 (13.6)	105 (14.2)	57 (7.5)	<0.001	0.849	0.002	<0.001
>9 h/day	147 (44.3)	330 (44.6)	457 (60.4)	<0.001	0.947	<0.001	<0.001
Weigh change in past year, *n* (%)	*n* = 330	*n* = 738	*n* = 741				
Increase	83 (25.2)	130 (17.6)	133 (17.9)	0.009	0.005	0.008	0.892
Decrease	58 (17.6)	95 (12.9)	133 (17.9)	0.017	0.047	0.931	0.008
No change	189 (57.3)	513 (69.5)	475 (64.1)	<0.001	<0.001	0.035	0.027
Family history of diabetes, *n* (%)	28/343 (8.2)	159/754 (21.1)	172/818 (21.0)	<0.001	<0.001	<0.001	1.000
Family history of hypertension, *n* (%)	119/343 (34.7)	354/754 (46.9)	371/818 (45.4)	<0.001	<0.001	0.001	0.544
Family history of dyslipidemia, *n* (%)	17/343 (5.0)	162/754 (21.5)	169/818 (20.7)	<0.001	<0.001	<0.001	0.710
Waist circumference, cm	87.7 ± 8.9	84.1 ± 9.7	87.2 ± 10.1	<0.001	<0.001	0.460	<0.001
Systolic blood pressure, mmHg	133.3 ± 21.6	130.2 ± 18.8	125.7 ± 17.7	<0.001	0.022	<0.001	<0.001
Diastolic blood pressure, mmHg	82.2 ± 10.0	78.8 ± 10.6	77.0 ± 9.7	<0.001	<0.001	<0.001	<0.001
Serum triglycerides, mmol/L	1.9 ± 1.3	1.8 ± 0.9	1.7 ± 1.0	0.005	1.146	0.003	0.032
Serum HDL-C, mmol/L	1.4 ± 0.3	1.4 ± 0.3	1.4 ± 0.5	0.125			
FPG, mmol/L	5.5 ± 1.9	5.9 ± 2.0	5.8 ± 1.5	0.001	0.001	0.001	0.360

Data are presented as the mean ± SD or *n* (%).

FPG, fasting plasma glucose; HDL-C, high-density lipoprotein cholesterol; MetS, metabolic syndrome.

After comparative analysis, logistic regression was performed for the evaluation of possible risk factors (Table 5). Age, rural area, half-meat and half-vegetable dietary style, no diet control, weight increase in the past year and hypertension in the family history were found to be important risk factors for MetS.

**Table 5:** Results of logistic regression analysis.

IDF					
Variable	Category	Ref.	OR	95% CI	*p*-value
Age (years)	50–59	1.000			
	60–69		1.831	1.444–2.323	<0.001
	>70		2.454	1.792–3.362	<0.001
Location	Urban	1.000			
	Rural		1.528	1.090–2.142	0.014
	Suburban		1.092	0.859–1.387	0.473
Education level	College and above	1.000			
	Middle school		1.306	0.909–1.876	0.149
	Elementary school and below		1.425	0.934–2.175	0.100
Family income	>¥30,000 (≥$4,200)/year	1.000			
	¥10,000–30,000 ($1,400–4,200)/year		1.122	0.871–1.447	0.372
	≤¥10,000 (≤$1,400)/year		1.105	0.816–1.495	0.520
Menstruation delay	No	1.000			
	Yes		1.042	0.665–1.631	0.858
Dietary style	Meat-based		1.822	0.909–3.649	0.091
	Half and half		1.400	1.107–1.770	0.005
	Vegetable-based	1.000			
Dietary salt consumption	High-salt		1.064	0.784–1.445	0.690
	Normal-salt		0.866	0.684–1.096	0.231
	Low-salt	1.000			
Dietary meat consumption	Fatty meats		1.141	0.550–2.368	0.723
	Moderately fatty meat		0.878	0.703–1.096	0.250
	Lean meats	1.000			
Diet control	No	1.000			
	Yes		1.555	1.242–1.947	<0.001
Sleep duration	≤6 h	1.000			
	6–8 h		1.198	0.890–1.613	0.234
	>8 h		1.303	0.943–1.801	0.109
Sedentary time	≤7 h/day	1.000			
	7–9 h/day		1.043	0.732–1.486	0.815
	>9 h/day		1.112	0.883–1.401	0.368
Weight change in the past year	No change	1.000			
	Increase		1.725	1.312–2.268	<0.001
	Decrease		1.169	0.873–1.566	0.293
Family history of diabetes	No	1.000			
	Yes		1.117	0.847–1.473	0.434
Family history of hypertension	No	1.000			
	Yes		1.854	1.483–2.319	<0.001
Family history of dyslipidemia	No	1.000			
	Yes		1.060	0.794–1.417	0.691

## 4 Discussion

As shown in Table 5, our study revealed that age, rural area, half-meat and half-vegetable dietary style, diet control, weight increase in the past year and hypertension in the family history were important risk factors for MetS. These findings are consistent with those of previous studies (5, 21, 22). According to IDF criteria, MetS prevalence was greater in rural region females than in urban and suburban region females 53.6% *vs.* 45.5 and 52.0%, (*p* = 0.003) (Table 2). Comparing the prevalence of single MetS components, central obesity and hypertension rates were highest in rural areas, but hyperglycemia and decreased HDL-C values were lowest. In urban females, hypertriglyceridemia occurred most frequently but the central obesity prevalence was lowest. Suburban females had the most decreased HDL-C values and highest prevalence of hyperglycemia, but the lowest hypertriglyceridemia and hypertension rates (Table 3). Historically, most Chinese studies have previously reported higher MetS prevalence in urban residents than in rural residents (4, 23–26). However, the results of a 2013–2014 nationwide MetS epidemiology study and a more recent Taiwanese study showed higher prevalence of MetS in rural than in urban populations (27, 28), which is in agreement with the present findings. Since the prevalence of MetS in the present study was influenced by the degree of urbanization, with rural and suburban regions experiencing significant increases, area specific factors like rapid economic development, dietary transitions and lifestyle modifications might be the reasons. With economic development, the food supplied to rural people became apparently different from traditional food, such as animal fat, simple carbohydrates (particularly fructose) and energy-dense foods (e.g., sugary drinks, biscuits) (29–31).

However, the findings that a half-meat, half-vegetable dietary pattern and dietary control were risk factors for MetS, remain difficult fully to explain. Previous studies have suggested that both of these factors may help prevent or improve MetS, but this hypothesis requires verification in prospective or retrospective population-based studies. Given that the present study was cross-sectional, it could not confirm any causal or temporal relationships. It is possible that participants with MetS began dietary control and adjusted their diet composition after being diagnosed, which may have led to these factors appearing as potential risk factors in the cross-sectional analysis. Unfortunately, this study did not collect information on the timing of dietary adjustments or when individuals started dietary control, preventing us from performing further analyses to clarify the issue. Another factor might be that with economic development the awareness of health management became higher, which might have resulted in stabilization and a gradual decline of obesity and MetS prevalence in Chinese urban areas (32). Therefore, the higher prevalence of MetS in rural women may be a historical phenomenon in the economic developing process, which might disappear when the economy and awareness of health are better developed.

The influence of economic development on dietary shifts and health awareness is important since there is epidemiological evidence of a link between income and MetS (33–35). However, in the regression analysis of the present study there was no significant correlation between family income and MetS. An explanation might be that during the development, especially of suburban areas, affordable fast food chain shops were established rapidly and the shift from traditional meals to fast food was income, independently taking place and therefore not an individual but rather a regional factor. Hypertension in the family history may suggest genetic factors for MetS (21, 36–38) and it is proposed that individuals with hypertension in the family history should focus on timely lifestyle changes to reduce the MetS risk, especially when weight increase occurs rapidly. However, these factors might mainly play a role and be regulated by health awareness, which might be attributed to the educational level, which is higher in urban adults compared to those in rural habitats.

Activity habits and awareness of health management might be improved as advocated by Japanese and Mexican researchers (31, 39). Also, in China, prevention-control measures have been established in the past 10 years but need further improvements (27, 30). It is noteworthy that there have been recent studies about successful public health interventions in Africa that reduced the incidence of MetS through community-based lifestyle interventions (40–42).

The limitations of the present study were that for certain small effect sizes or in analyses involving specific subgroups with limited sample sizes (e.g., women of a specific age group in a particular region), non-significant results may be influenced by limited statistical power. As we did not exclude participants who had already initiated dietary management due to pre-existing chronic conditions, it was found during data analysis that the prevalence of MetS was actually higher among individuals who reported engaging in dietary management compared to those who did not. Moreover, dietary management emerged as a significant risk factor for MetS in the regression analysis. This finding is counterintuitive and prompted further reflection. One plausible explanation is that participants may have adopted dietary management in response to a prior diagnosis of a chronic disease. Given the cross-sectional nature of our study, it was not possible to establish the temporal sequence of exposure and outcome, which could have led to this unexpected association. The root of this limitation lies in the lack of detailed survey items capturing the reasons for dietary management. As a result, we were unable to exclude or appropriately classify participants who initiated dietary management due to prior health conditions during analysis, which is a methodological shortcoming. Furthermore, excluding all participants with chronic diseases who reported dietary management could substantially reduce the sample size and affect the representativeness of our findings, as the proportion of individuals aged ≥50 years without chronic diseases was relatively low. In future studies with similar designs, we plan to incorporate specific questions regarding the motivation for dietary management (e.g., whether it was initiated due to a diagnosed illness) to allow more accurate data classification and interpretation. We also intend to develop analytical strategies that better elucidate the relationship between dietary management and the occurrence of MetS.

## 5 Conclusion

The present study highlights a significantly higher prevalence of MetS among rural women aged ≥50 years in northwest China compared to their urban and suburban counterparts. Key risk factors identified include an older age, rural residence, dietary habits and weight gain in the past year. The elevated MetS rates among rural women appear to be linked to distinct socioeconomic and lifestyle characteristics, including limited health literacy and reduced engagement in dietary control. These findings emphasize the urgent need for region-specific public health strategies targeting metabolic health, especially among aging rural female populations. Addressing health disparities through education, early screening and lifestyle interventions will be crucial for mitigating MetS-related risks and promoting equitable health outcomes across diverse populations in China.

## Data Availability

The raw data supporting the conclusions of this article will be made available by the authors, without undue reservation.

## References

[1] Mottillo S Filion KB Genest J Joseph L Pilote L Poirier P. The metabolic syndrome and cardiovascular risk a systematic review and meta-analysis. J Am Coll Cardiol. (2010) 56:1113–32. doi: 10.1016/j.jacc.2010.05.034, PMID: 20863953

[2] Dragsbæk K Neergaard JS Laursen JM Hansen HB Christiansen C Beck-Nielsen H. Metabolic syndrome and subsequent risk of type 2 diabetes and cardiovascular disease in elderly women: challenging the current definition. Medicine (Baltimore). (2016) 95:e4806-e. doi: 10.1097/MD.0000000000004806, PMID: 27603394 PMC5023917

[3] Novak M Björck L Welin L Welin C Manhem K Rosengren A. Gender differences in the prevalence of metabolic syndrome in 50-year-old Swedish men and women with hypertension born in 1953. J Hum Hypertens. (2013) 27:56–61. doi: 10.1038/jhh.2011.106, PMID: 22129609

[4] Gu D Reynolds K Wu X Chen J Duan X Reynolds RF. Prevalence of the metabolic syndrome and overweight among adults in China. Lancet. (2005) 365:1398–405. doi: 10.1016/s0140-6736(05)66375-1, PMID: 15836888

[5] Stefanska A Bergmann K Sypniewska G. Metabolic syndrome and menopause: pathophysiology, clinical and diagnostic significance. Adv Clin Chem. (2015) 72:1–75. doi: 10.1016/bs.acc.2015.07.001 26471080

[6] Perry A Wang X Goldberg R Ross R Jackson L. Androgenic sex steroids contribute to metabolic risk beyond intra-abdominal fat in overweight/obese black and white women. Obesity (Silver Spring). (2013) 21:1618–24. doi: 10.1002/oby.20204, PMID: 23670917

[7] Shakir YA Samsioe G Nyberg P Lidfeldt J Nerbrand C Agardh CD. Do sex hormones influence features of the metabolic syndrome in middle-aged women? A population-based study of Swedish women: the women's health in the Lund area (Whila) study. Fertil Steril. (2007) 88:163–71. doi: 10.1016/j.fertnstert.2006.11.111, PMID: 17383645

[8] Choe SA Yoon NH Yoo S Kim H. Gender-differences in predictors for time to metabolic syndrome resolution: a secondary analysis of a randomized controlled trial study. PLoS One. (2020) 15:e0234035. doi: 10.1371/journal.pone.0234035, PMID: 32584834 PMC7316247

[9] Xiao J Wu C-L Gao Y-X Wang S-L Wang L Lu Q-Y. Prevalence of metabolic syndrome and its risk factors among rural adults in Nantong, China. Sci Rep. (2016) 6:38089. doi: 10.1038/srep38089, PMID: 27901076 PMC5128865

[10] Guo H Gao X Ma R Liu J Ding Y Zhang M. Prevalence of metabolic syndrome and its associated factors among multi-ethnic adults in rural areas in Xinjiang, China. Sci Rep. (2017) 7:17643. doi: 10.1038/s41598-017-17870-5, PMID: 29247195 PMC5732195

[11] Han B Chen Y Cheng J Li Q Zhu C Chen Y. Comparison of the prevalence of metabolic disease between two types of urbanization in China. Front Endocrinol. (2018) 9:665. doi: 10.3389/fendo.2018.00665, PMID: 30483219 PMC6240687

[12] Wang GR Li L Pan YH Tian GD Lin WL Li Z. Prevalence of metabolic syndrome among Urban Community residents in China. BMC Public Health. (2013) 13:599. doi: 10.1186/1471-2458-13-599, PMID: 23786855 PMC3734094

[13] Van Wormer JJ Boucher JL Sidebottom AC Sillah A Knickelbine T. Lifestyle changes and prevention of metabolic syndrome in the heart of New Ulm project. Prev Med Rep. (2017) 6:242–5. doi: 10.1016/j.pmedr.2017.03.018, PMID: 28377851 PMC5377429

[14] Garralda-Del-Villar M Carlos-Chillerón S Diaz-Gutierrez J Ruiz-Canela M Gea A Martínez-González MA. Healthy lifestyle and incidence of metabolic syndrome in the sun cohort. Nutrients. (2018) 11:65. doi: 10.3390/nu11010065, PMID: 30598006 PMC6357191

[15] Kataria I Chadha R Pathak R. Dietary and lifestyle modification in metabolic syndrome: a review of randomized control trials in different population groups. Rev Health Care. (2013) 4:22. doi: 10.7175/rhc.v4i4.667

[16] Wang Z Su L. Prevalence of metabolic syndrome in urban area of Fuzhou. Chin J Hypertens. (2007) 15:128–31. doi: 10.3969/j.issn.1673-7245.2007.02.013

[17] Ming J Xu S Xie X Wang Y Yao X Jia A. Diabetes prevalence and its comorbidities in people aged 50 years and above in Xi'an: a community-based survey. Chin J Diabetes Mellit. (2018) 10:531–6. doi: 10.3760/cma.j.issn.1674-5809.2018.08.007

[18] National Bureau of Statistics of China. Regulation on classification of urban and rural areas for statistics. (2008). Available online at: https://www.stats.gov.cn/xxgk/tjbz/gjtjbz/201310/t20131031_1758903.html (Accessed April 24, 2024).

[19] Chinese Diabetes Society. Guideline for the prevention and treatment of type 2 diabetes mellitus in China (2013 edition). Chin J Diabetes Mellit. (2013) 6:447–98. doi: 10.3760/cma.j.issn.1674-5809.2014.07.004

[20] International Diabetes Federation. The IDF consensus worldwide definition of the metabolic syndrome. (2006). Available online at: https://idf.org/media/uploads/2023/05/attachments-30.pdf (Accessed April 24, 2024).

[21] Ranasinghe P Cooray DN Jayawardena R Katulanda P. The influence of family history of hypertension on disease prevalence and associated metabolic risk factors among Sri Lankan adults. BMC Public Health. (2015) 15:576. doi: 10.1186/s12889-015-1927-7, PMID: 26092387 PMC4475303

[22] Zhao Y Yan H Yang R Li Q Dang S Wang Y. Prevalence and determinants of metabolic syndrome among adults in a rural area of Northwest China. PLoS One. (2014) 9:e91578. doi: 10.1371/journal.pone.0091578, PMID: 24614618 PMC3948893

[23] Weng X Liu Y Ma J Wang W Yang G Caballero B. An urban-rural comparison of the prevalence of the metabolic syndrome in eastern China. Public Health Nutr. (2007) 10:131–6. doi: 10.1017/S1368980007226023, PMID: 17261221

[24] Zuo H Shi Z Hu X Wu M Guo Z Hussain A. Prevalence of metabolic syndrome and factors associated with its components in Chinese adults. Metabolism. (2009) 58:1102–8. doi: 10.1016/j.metabol.2009.04.008, PMID: 19481771

[25] Zhao J Pang ZC Zhang L Gao WG Wang SJ Ning F. Prevalence of metabolic syndrome in rural and urban Chinese population in Qingdao. J Endocrinol Investig. (2011) 34:444–8. doi: 10.1007/BF03346711 21270510

[26] Lao XQ Zhang YH Wong MC Xu YJ Xu HF Nie SP. The prevalence of metabolic syndrome and cardiovascular risk factors in adults in southern China. BMC Public Health. (2012) 12:64. doi: 10.1186/1471-2458-12-64 22264227 PMC3293058

[27] Lan Y Mai Z Zhou S Liu Y Li S Zhao Z. Prevalence of metabolic syndrome in China: an up-dated cross-sectional study. PLoS One. (2018) 13:e0196012. doi: 10.1371/journal.pone.0196012, PMID: 29668762 PMC5906019

[28] Wang WS Wahlqvist ML Hsu CC Chang HY Chang WC Chen CC. Age- and gender-specific population attributable risks of metabolic disorders on all-cause and cardiovascular mortality in Taiwan. BMC Public Health. (2012) 12:111. doi: 10.1186/1471-2458-12-111, PMID: 22321049 PMC3305485

[29] Misra A Khurana L. Obesity and the metabolic syndrome in developing countries. J Clin Endocrinol Metab. (2008) 93:S9–S30. doi: 10.1210/jc.2008-1595 18987276

[30] Hu FB Liu Y Willett WC. Preventing chronic diseases by promoting healthy diet and lifestyle: public policy implications for China. Obes Rev. (2011) 12:552–9. doi: 10.1111/j.1467-789X.2011.00863.x, PMID: 21366840

[31] Yoneda M Kobuke K. A 50-year history of the health impacts of westernization on the lifestyle of Japanese Americans: a focus on the Hawaii-Los Angeles-Hiroshima study. J Diabetes Investig. (2020) 11:1382–7. doi: 10.1111/jdi.13278, PMID: 32311224 PMC7610102

[32] Beltran- Sanchez H Harhay MO Harhay MM McElligott S. Prevalence and trends of metabolic syndrome in the adult U.S. population, 1999-2010. J Am Coll Cardiol. (2013) 62:697–703. doi: 10.1016/j.jacc.2013.05.064, PMID: 23810877 PMC3756561

[33] Braveman P Gottlieb L. The social determinants of health: it's time to consider the causes of the causes. Public Health Rep. (2014) 129:19–31. doi: 10.1177/00333549141291S206, PMID: 24385661 PMC3863696

[34] Moore JX Chaudhary N Akinyemiju T. Metabolic syndrome prevalence by race/ethnicity and sex in the United States, National Health and nutrition examination survey, 1988-2012. Prev Chronic Dis. (2017) 14:E24. doi: 10.5888/pcd14.160287, PMID: 28301314 PMC5364735

[35] Ye Q Wang Z Deng T Lou Q Wu H Tang W. Association of socioeconomic status with metabolic syndrome and its components among adult population: a community-based cross-sectional study in Nanjing municipality of China. BMJ Open. (2023) 13:e074059. doi: 10.1136/bmjopen-2023-074059, PMID: 37844993 PMC10582845

[36] Li P Tao Y Su H Cheng X Yu H Wu Q. Metabolic syndrome tendency of healthy young offsprings of hypertension patients. Chin J Hypertens. (2008) 16:845–6. doi: 10.3969/j.issn.1673-7245.2008.09.021

[37] Harrison TA Hindorff LA Kim H Wines RCM Bowen DJ McGrath BB. Family history of diabetes as a potential public health tool. Am J Prev Med. (2003) 24:152–9. doi: 10.1016/s0749-3797(02)00588-3 12568821

[38] Yoon PW Scheuner MT Peterson-Oehlke KL Gwinn M Faucett A K MJ. Can family history be used as a tool for public health and preventive medicine? Genet Med. (2002) 4:304–10. doi: 10.1097/00125817-200207000-00009 12172397

[39] Levaillant M Lièvre G Baert G. Ending diabetes in Mexico. Lancet. (2019) 394:467–8. doi: 10.1016/s0140-6736(19)31662-9, PMID: 31402023

[40] Mamun A Kitzman H Dodgen L. Reducing metabolic syndrome through a community-based lifestyle intervention in African American women. Nutr Metab Cardiovasc Dis. (2020) 30:1785–94. doi: 10.1016/j.numecd.2020.06.005, PMID: 32605881 PMC7494631

[41] Lubogo D Wamani H Mayega RW Orach CG. Effects of nutrition education, physical activity and motivational interviewing interventions on metabolic syndrome among females of reproductive age in Wakiso District, Central Uganda: a randomised parallel-group trial. BMC Public Health. (2025) 25:790. doi: 10.1186/s12889-025-21936-9, PMID: 40011877 PMC11866849

[42] Okube OT Kimani S Mirie W. Community-based lifestyle intervention improves metabolic syndrome and related markers among Kenyan adults. J Diabetes Metab Disord. (2022) 21:607–21. doi: 10.1007/s40200-022-01023-1, PMID: 35673420 PMC9167372

